# Factors influencing delirium nursing competency among nurses in integrated nursing care wards in South Korea: a cross-sectional study

**DOI:** 10.7586/jkbns.25.022

**Published:** 2025-05-27

**Authors:** Jeeyoung Yeon, Gisoo Shin

**Affiliations:** College of Nursing, Chung-Ang University, Seoul, Korea

**Keywords:** Delirium, Knowledge, Emotional intelligence, Nurses, Nursing care

## Abstract

**Purpose:**

This study investigated the levels of delirium knowledge, delirium nursing-related stress, emotional intelligence, and delirium nursing competency among nurses in integrated nursing care wards, and aimed to identify factors influencing their competency.

**Methods:**

A descriptive survey design was employed, and data were collected from 184 nurses working in integrated nursing care wards at general hospitals located in Seoul and Daejeon, South Korea. The research instruments included measures of delirium knowledge, delirium nursing-related stress, emotional intelligence, and delirium nursing competency.

**Results:**

Delirium knowledge, emotional intelligence, and experience with delirium education were significant influencing factors, explaining 12% of the variance on delirium nursing competency.

**Conclusion:**

Based on these findings, it is recommended that future research within the field of nursing should explore a broader range of variables that may influence delirium nursing competency, particularly among nurses working in integrated nursing settings. In addition, from the perspective of foundational nursing education, it is necessary to conduct experimental studies and develop targeted educational programs aimed at reaching a deeper understanding of the various mechanisms underlying delirium.

## INTRODUCTION

Delirium, derived from the Latin term deliria, meaning “to go off the furrow,” refers to an acute and fluctuating disturbance in consciousness, orientation, memory, cognition, perception, and behavior [1]. This condition is frequently observed among patients in neurological intensive care units and occurs more frequently in older adults, post-surgical patients, and those with comorbid brain or chronic systemic conditions [2]. Early detection and timely management of delirium are crucial, as it is often reversible with appropriate intervention [1,2].

Older adults are particularly vulnerable to delirium due to diminished homeostatic regulation, sensory impairments, high prevalence of chronic illnesses, reduced resilience to acute stressors, sleep deprivation, and psychosocial stressors such as bereavement or social isolation [3]. Delirium in the elderly is often misdiagnosed as dementia or depression, leading to delayed treatment, which may result in stupor, coma, or even death in severe cases [4]. The pathophysiology of delirium involves disruptions in cerebral metabolism, decreased synthesis of acetylcholine, neurotransmitter imbalances, stress-related changes in brain metabolism, and right hemisphere dysfunction [1,2]. Neurotransmitter imbalances, particularly involving acetylcholine, dopamine, serotonin, gamma-amnionobutyric acid, glutamate, norepinephrine, opioids, somatostatin, beta-endorphin, and cortisol identified as key contributors to the development of delirium [5]. Delirium symptoms categorized into cognitive disturbances, psychiatric manifestations, sleep-wake cycle disruptions, and neurological abnormalities. These symptoms often intensify in the evening and tend to fluctuate significantly throughout the day [1,2,5].

In elderly patients, delirium may manifest through disorientation and wandering behaviors, such as pulling out intravenous lines or attempting to leave the hospital room, which can lead to falls [3]. Moreover, delirium in older adults recognized as an independent risk factor for increased mortality [4]. South Korea has projected to become a super-aged society by 2025, with individuals aged 65 and older comprising over 20% of the population, and this figure is expected to exceed 40% by 2050 [6]. In response, the government has implemented an integrated nursing care (INC) model to alleviate caregiver burden and improve patient safety amid rising chronic disease prevalence and increased family satisfaction. This model allows professional nurses and nursing assistants to deliver comprehensive care in place of informal caregivers, thereby enhancing direct nursing time, improving patient satisfaction, and yielding better health outcomes [7]. With the progression toward an aging society, the hospitalization rate of elderly patients who are at elevated risk for delirium is steadily increasing [4,6]. INC wards, which allow patients to receive nursing services without a caregiver present, see a higher admission rate among older adults, as families seek to alleviate the burden of caregiving [6,7]. As such, this ward presents a high-risk environment for the development of delirium [4]. For nurses working in such settings, possessing sufficient knowledge about delirium is essential for the early recognition of symptoms and the provision of timely and appropriate interventions. This is particularly important because delirium is an acute mental status change that can deteriorate rapidly, requiring nurses to make accurate judgments and respond effectively based on a solid understanding of the condition [2]. Furthermore, nursing with delirium patients can be highly stressful for nurses due to unpredictable behaviors, aggression, and fluctuating levels of consciousness [8]. In addition, nurses in INC wards often face higher patient-to-nurse ratios and the absence of family caregivers, which results in greater occupational stress compared to their counterparts in general wards or intensive care units [9]. Most critically, the lack of epidemiological data on the incidence of delirium in INC wards and the absence of standardized criteria for classifying its severity mean that patients with delirium are admitted to these wards without specific restrictions, thereby increasing the demand for nursing care in this context [10]. To address this issue, the 2024 revision of the service guidelines introduced resolute units for critically ill patients with delirium [11].

However, INC wards nurses found to lack both the skills to apply clinical guidelines, and the techniques required for proper delirium assessment [9,10]. Prior research has established a strong association between nurses’ knowledge of delirium and their competency in providing effective nursing care, with those possessing higher knowledge demonstrating greater proficiency [12]. Furthermore, delirium nursing care is not a mandatory component of nursing education in INC wards. Many nurses report not having received formal education on the subject, and delirium knowledge levels remain comparable to those reported a decade ago. This delirium knowledge deficit contributes significantly to nurses' stress when caring for delirious patients [9].

Emotional intelligence plays a crucial role in alleviating the stress experienced by nurses, particularly in high-demand clinical environments. It was defined as the ability to perceive, understand, regulate, and effectively utilize emotions [13]. Nurses with high emotional intelligence are better equipped to comprehend the confusion experienced by delirious patients, remain composed in the face of aggressive behaviors, and foster a stable and therapeutic care environment. This competency not only facilitates positive interactions with patients but also enhances communication and rapport with family members. Emotional intelligence contributes directly to patient safety and treatment outcomes in INC wards [14].

Delirium nursing competency is of particular importance for nurses in INC wards settings due to both the nature of these wards and the characteristics of patients with delirium. INC wards operate under a model in which nurses provide comprehensive care and daily living support without the presence of family caregivers. Given that delirium is strongly associated with cognitive decline, increased risk of injury, prolonged hospitalization, and higher mortality, a nurse’s ability to detect and manage delirium early can significantly influence patient outcomes [6,7]. Previous study has shown that nurses who receive delirium-specific education are more capable of recognizing symptoms promptly and responding effectively, which in turn improves recovery rates and overall quality of life for patients [15]. Moreover, because delirium is often dismissed as a temporary state of confusion, it requires a prominent level of clinical sensitivity to subtle changes in patient condition. Thus, delirium nursing competency encompasses more than theoretical knowledge; it involves a practice-oriented, integrative skill set that enables nurses to respond swiftly and appropriately in complex clinical scenarios [16].

Therefore, knowledge of delirium, the ability to manage related stress, emotional intelligence, and practical delirium nursing competency are interrelated core elements in the delivery of high-quality, patient-centered care in INC wards. This study aims to investigate the levels of delirium knowledge, delirium nursing-related stress, emotional intelligence, and delirium nursing competency among nurses in INC wards, and to identify factors influencing their competency. The findings are expected to provide foundational data for the development of targeted interventions aimed at enhancing delirium nursing competency in these settings.

## METHODS

### 1. Study Design

This descriptive correlational study aimed to examine the levels of delirium knowledge, delirium nursing-related stress, emotional intelligence, and delirium nursing competency among nurses working in INC wards and to identify factors influencing their competency in providing delirium nursing care.

### 2. Participants

The participants were nurses working in INC wards at general hospitals located in Seoul and Daejeon, South Korea. Based on previous studies indicating that newly appointed nurses require approximately six months to effectively acquire necessary clinical skills and knowledge, only nurses with more than six months of experience in INC wards and those with prior experience in caring for patients with delirium were included. Nurse managers were excluded from the study, as they do not provide direct patient care [17].

Sample size calculations using G*Power 3.1.9.7 [18] indicated that a minimum of 166 participants were required for a multiple regression analysis with an effect size of .15, alpha level of .05, power of .90, and 14 predictors. Considering an approximate dropout rate of 15%, the final target sample size was 191. Of the 191 distributed questionnaires, 184 were returned with complete responses and used for analysis.

### 3. Instruments

All instruments employed in this study were used with the original authors' permission.

#### 1) Delirium knowledge

Delirium knowledge was measured using a tool developed by Lee et al. [19] consisting of 45 items across three subscales: causes (10 items), symptoms (20 items), and nursing management (15 items). Each item was scored as 1 (correct) or 0 (incorrect/unknown), with a total score of 45. Higher scores indicated greater knowledge. The Cronbach’s α for this study was .83.

#### 2) Delirium nursing-related stress

Delirium nursing-related stress was assessed using a tool developed by Park and Gu [20]. This 20-item instrument measured perceived stress on a scale of 0 ("not confident") to 100 ("very confident") for each item, with higher scores indicating greater stress. The Cronbach’s α in this study was .94.

#### 3) Emotional intelligence

Emotional intelligence was measured using the instrument developed by Wong and Law [21]. The 16 items covered four domains: self-emotional appraisal, others’ emotional appraisal, regulation of emotion, and use of emotion. Each item was rated on a 7-point Likert scale (1 = strongly disagree, 7 = strongly agree). The Cronbach’s α in this study was .94.

#### 4) Delirium nursing competency

Delirium nursing competency was measured using a tool developed by Roh [22], consisting of 24 items covering six domains: algorithm management, prevention, communication, nursing care, evaluation, and collaboration. Responses were rated on a 4-point Likert scale (1 = not competent, 4 = very competent), with higher scores indicating greater competency. The Cronbach’s α was .94.

### 4. Data collection

Data were collected from April 11 to June 28, 2024, across one general hospital in Seoul and three in Daejeon, South Korea. Prior to data collection, the study’s objectives and procedures were explained to the nursing departments and written approval was obtained. Participation was voluntary and preceded by informed consent. Completed questionnaires were sealed in individual envelopes and deposited in a secure collection box in each department. Participants received a small token of appreciation.

### 5. Data analysis

Data were analyzed using SPSS version 29.0 (IBM Corp., Armonk, NY, USA). Descriptive statistics and frequency analyses were used to summarize demographic characteristics and variable distributions. Differences in variables by general and delirium-related characteristics were analyzed using t-tests and one-way analysis of variance, with post-hoc comparisons via Duncan’s test. Pearson correlation coefficient was used to examine relationships among variables, and multiple linear regression was used to identify predictors of delirium nursing competency.

### 6. Ethical considerations

The Institutional Review Board of Chung-Ang University (IRB No. 1041078-20240131-HR-021) approved this study. All participants provided informed written consent after being assured of confidentiality, anonymity, voluntary participation, and the right to withdraw at any time without penalty. The collected data were coded for confidentiality and securely stored according to IRB guidelines.

## RESULTS

### 1. Characteristics of participants

Among the participants, 91 nurses (49.5%) were in their 20s, 61 (33.2%) in their 30s, and 32 (17.3%) were aged 40 or older. In terms of educational level, 157 (85.3%) held a bachelor's degree, 20 (10.9%) held an associate degree, and 7 (3.8%) held a master’s degree. The average total clinical career was 6.30 ± 5.88 years, and the average duration of career in the current department was 2.70 ± 2.22 years. Regarding the availability of manual for delirium nursing care in the ward, 99 participants (53.8%) responded affirmatively, while 85 (46.2%) reported the absence of such manual for delirium nursing care. A total of 111 participants (60.3%) had experience of education for delirium, with 70.3% citing "clinical experience" and 29.7% citing "continuing education" as the primary modes of instruction. All participants (97.8%) expressed a need for delirium education, and 74.5% reported experiencing conflict with caregivers of patients with delirium (Table 1).

### 2. Levels of delirium knowledge, delirium nursing-related stress, emotional intelligence, and delirium nursing competency

The mean score for delirium knowledge was 32.10 ± 4.80 out of 45, with the highest correct response rate in the subdomain of etiology and the lowest in the domain of nursing management. The average score for delirium nursing-related stress was 57.83 ± 18.15 out of 100, while the mean emotional intelligence score was 80.03 ± 12.50 out of 112. The mean score for delirium nursing competency was 68.87 ± 8.04 out of 96 (Table 2).

### 3. Differences in the levels of delirium knowledge, delirium nursing-related stress, emotional intelligence, and delirium nursing competency according to participants` characteristics

Delirium knowledge showed statistically significant differences based on age (F = 4.68, *p* = .010), position (t = −2.25, *p* = .026), total clinical career (F = 3.46, *p* = .033), experience with delirium education (t = 3.26, *p* = .001). Delirium nursing-related stress, however, did not significantly differ by age (F = 0.58, *p* = .561), educational level (F = 0.84, *p* = .434), position (t = −1.32, *p* = .095), total clinical carrier (F = 0.20, *p* = .820), career in the current department (F = 0.86, *p* = .426), third-shift work (t = −0.20, *p* = .840), presence manual for delirium nursing care (t = −1.22, *p* = .226), experience of education for delirium (t = −1.17, *p* = .242), perceived need for delirium education (t = 0.87, *p* = .384), or conflict with the caregiver of patients with delirium (t = 0.47, *p* = .638). Emotional intelligence varied significantly with age (F = 4.10, *p* = .018) and experience of education for delirium (t = −3.16, *p* = .002). Delirium nursing competency significantly differed only by experience of education for delirium (t = 2.09, *p* = .038) (Table 3).

### 4. Correlation among delirium knowledge, delirium nursing-related stress, emotional intelligence, and delirium nursing competency

There were significant positive correlations between delirium nursing competency and delirium knowledge (r = .24, *p* = .001), as well as between delirium nursing competency and emotional intelligence (r = .23, *p* = .001) (Table 4).

### 5. Factors influencing delirium nursing competency

To identify factors influencing delirium nursing competency, multiple regression analysis was conducted using variables that showed significant differences, specifically experience of education for delirium, need for delirium education, delirium knowledge, delirium nursing-related stress, and emotional intelligence as independent variables. The experience of education for delirium was treated as a dummy variable.

The regression model was statistically significant (F = 7.18, *p* < .001). The Durbin-Watson statistic was 1.94, indicating no violation of independence assumptions, and variance inflation factors were all below 10, suggesting no multi-collinearity concerns.

Significant predictors of delirium nursing competency included experience of education for delirium (β = 0.16, p = .031), delirium knowledge (β = 0.20, *p* = .006), and emotional intelligence (β = 0.27, *p* < .001). Among these, emotional intelligence had the strongest influence, followed by delirium knowledge and experience of education for delirium. The explanatory power of the model was 12% (adjusted R^2^ = 12%) (Table 5).

## DISCUSSION

This study investigated levels of delirium knowledge, delirium nursing-related stress, emotional intelligence, and delirium nursing competency among nurses working in INC wards across four general hospitals and examined the factors influencing their delirium nursing competency.

The mean score for delirium knowledge (32.10 out of 45) was consistent with previous studies conducted in tertiary hospitals using the same instrument [10], yet lower than scores reported in cancer specialty hospitals where nurses are more frequently exposed to delirium [23]. These discrepancies likely reflect institutional differences in delirium prevalence. The expansion of INC wards following the coronavirus disease 2019 pandemic has increased exposure to delirium education and manual [24]. However, delirium remains an underemphasized topic in required educational programs within these units. Compared to international study, the delirium knowledge level remains low, underscoring the need for tailored, mandatory education programs that align with the ward’s unique characteristics [15]. Among delirium knowledge subdomains, the highest scores were in delirium etiology, while the lowest were in nursing management findings consistent with prior research [19]. This suggests a gap between symptom recognition and appropriate intervention, highlighting the importance of strengthening training focused on nursing management of delirium.

The average delirium nursing-related stress score was 57.83 out of 100, comparable to or slightly lower than scores reported in tertiary hospitals [25]. Given the lower severity of cases in general hospitals, this outcome seems plausible. Nonetheless, unresolved stress related to delirium nursing care may lead to burnout and job dissatisfaction, emphasizing the need for psychological support and institutional interventions.

The average emotional intelligence score was 80.03 out of 112. Participants demonstrated stronger self-awareness but lower scores in emotional utilization and regulation, mirroring previous findings [26]. Given the close, continuous interaction with patients in this ward, the development of emotional intelligence particularly in regulation and application, is essential for effective therapeutic relationships [14]. Thus, educational programs focusing on emotional intelligence should be developed and aligned with institutional needs.

The mean delirium nursing competency score was 68.87 out of 96, slightly lower than that reported among intensive care unit (ICU) nurses [22], due to the higher incidence and risk of delirium in ICU. Notably, low subdomain scores in collaboration, communication, and evaluation suggest a connection with knowledge deficiencies and lack of confidence [15,26]. To enhance collaboration and communication among nurses in INC wards, educational programs must address both knowledge and emotional competencies [27]. This study also confirmed that nurses who had experience of education for delirium scored higher in nursing competency, consistent with prior research [22]. Education for delirium has been shown to reduce complications by improving nursing performance [16], and competency can be enhanced through repeated instruction. Hence, sustainable, and diverse educational strategies are needed.

Significant positive correlations were found among delirium knowledge, emotional intelligence, and delirium nursing competency, confirming that both delirium knowledge and emotional intelligence are key predictors. Experience of education for delirium also played a crucial role. Also, the findings of this study indicate that delirium knowledge and emotional intelligence are key factors influencing nurses’ competency in delirium nursing care in INC wards. Given that delirium is an acute cognitive disorder with rapid onset and fluctuating symptoms, early recognition and timely intervention are critical both of which are largely dependent on the nurse’s level of knowledge regarding delirium [9,19]. When nurses possess sufficient understanding of their causes, symptoms, and appropriate interventions, they are better equipped to deliver effective care, making delirium knowledge a core component of delirium nursing competency [10]. Furthermore, nurses with high emotional intelligence are more attuned to the nonverbal cues of patients with delirium, can respond with empathy, and play a significant role in building trust not only with patients but also with their families [7,12,14]. This highlights emotional intelligence as an essential attribute for nurses in integrated care settings [28]. Considering the unique environment of INC wards, where family members or caregivers are often not present, the role of the nurse as a primary support figure becomes even more crucial, especially in managing the confusion and anxiety experienced by patients with delirium [13,27]. Therefore, an integrated educational program that enhances both delirium knowledge and emotional intelligence is essential to improve nurses’ competency in delirium nursing care.

Notably, stress related to delirium did not have a significant impact on delirium nursing competency. This may be attributed to the fact that the participants were employed at general hospitals, where the severity of patient conditions and the proportion of patients with delirium may be relatively lower compared to those in tertiary care settings [29].

This study has limitations in generalizability due to the use of convenience sampling from general hospitals in specific regions. Nevertheless, it offers valuable insights by identifying predictors of delirium nursing competency and comparing them with prior findings, thereby reinforcing the importance of delirium nursing care within integrated nursing care.

## CONCLUSION

This descriptive study analyzed data from 184 nurses working in INC wards at four general hospitals located in Seoul and Daejeon, South Korea. It aimed to assess their levels of delirium knowledge, delirium nursing–related stress, emotional intelligence, and delirium nursing competency, and to identify factors influencing delirium nursing competency. The findings revealed that delirium knowledge, emotional intelligence, and experience of education for delirium were significant influencing factors, explaining 12% of the variance in delirium nursing competency. Based on these findings, it is recommended that future research within the field of nursing should continue to explore a broader range of variables that may influence delirium nursing competency, particularly among nurses working in integrated care settings. In addition, the delirium nursing competency of nurses working in INC wards is expected to have significant academic and clinical implications for the Korean biological nursing science. In particular, nurses' delirium experiences and case-based insights can inform the development of educational curricula and policy recommendations promoted by society, contributing to the integration of academic theory with clinical practice for delirium patients. These practical competencies are anticipated to serve as a catalyst in shifting the society’s research agenda toward a more clinical orientation, playing a key role in bridging the gap between academic advancement and practical application.

## Figures and Tables

**Table 1.** General Characteristics of Participants (N = 184)

Characteristics	n (%)	M ± SD
Age (yr)		
< 30	91 (49.5)	
30~39	61 (33.2)	
≥ 40	32 (17.3)	
Educational level		
Associate’s degree	20 (10.9)	
Bachelor’s degree	157 (85.3)	
≥ Master’s degree	7 (3.8)	
Position		
Staff nurse	169 (91.8)	
Charge nurse	15 (8.2)	
Total clinical career (yr)		6.30 ± 5.88
< 5	102 (55.5)	
5~9	40 (21.7)	
≥ 10	42 (22.8)	
Current department career (yr)		2.70 ± 2.22
< 2	80 (43.5)	
2~4	67 (36.4)	
≥ 5	37 (20.1)	
Third-shift work		
Yes	154 (83.7)	
No	30 (16.3)	
Manual for delirium nursing care		
Yes	99 (53.8)	
No	85 (46.2)	
Experience of education about delirium		
Yes	111 (60.3)	
Education route		
Clinical experience	78 (70.3)	
Continuing education	33 (29.7)	
No	73 (39.7)	
Need for delirium education		
Necessary	180 (97.8)	
Not necessary	4 (2.2)	
Conflict with the caregiver of a patient with delirium		
Yes	137 (74.5)	
No	47 (25.5)	

M = Mean; SD = Standard deviation.

**Table 2.** Levels of Delirium Knowledge, Delirium Nursing-related Stress, Emotional Intelligence, and Delirium Nursing Competency (N = 184)

Variables	Range	Min~Max	M ± SD
Delirium knowledge			
Etiology	0~10	1~10	8.97 ± 1.44
Symptoms	0~20	5~20	13.64 ± 2.42
Nursing management	0~15	0~15	9.49 ± 2.68
Total	0~45	11~45	32.10 ± 4.80
Delirium nursing-related stress	0~100	0~100	57.83 ± 18.15
Emotional intelligence			
Self-emotional appraisal	4~28	12~28	20.93 ± 3.62
Others’ emotional appraisal	4~28	8~28	20.39 ± 3.81
Regulation of emotion	4~28	7~28	19.03 ± 4.04
Use of emotion	4~28	5~28	19.68 ± 3.75
Total	16~112	41~112	80.03 ± 12.50
Delirium nursing competency			
Manage algorithm	6~24	6~18	13.67 ± 2.76
Prevention	5~20	7~20	15.36 ± 2.40
Communication	4~16	5~16	11.62 ± 2.09
Nursing management	4~16	8~16	12.98 ± 1.91
Assessment	3~12	5~12	9.11 ± 1.48
Collaboration	2~8	2~8	6.13 ± 1.07
Total	24~96	48~90	68.87 ± 8.04

M = Mean; SD = Standard deviation; Min = Minimum; Max = Maximum.

**Table 3.** Differences in the Levels of Delirium Knowledge, Delirium Nursing-related Stress, Emotional Intelligence, and Delirium Nursing Competency According to Participant’s Characteristics (N = 184)

Characteristics		Delirium knowledge		Delirium nursing-related stress		Emotional intelligence		Delirium nursing competency	
		M ± SD	t or F (*p)*	M ± SD	t or F (*p)*	M ± SD	t or F (*p)*	M ± SD	t or F (*p)*
Age (yr)^†^	< 30^a^	31.05 ± 4.52	4.68 (.010)	57.02 ± 17.23	0.58 (.561)	5.04 ± 0.75	4.10 (.018)	2.85 ± 0.33	0.33 (.722)
	30~39^b^	32.87 ± 5.07	a < c	59.85 ± 19.02		4.81 ± 0.91	a < c	2.89 ± 0.33	
	≥ 40^c^	33.59 ± 4.45		56.31 ± 19.24		5.28 ± 0.48		2.89±0.47	
Educational level	Associate’s degree	30.90 ± 6.08	2.35 (.099)	55.04 ± 22.96	0.84 (.434)	4.80 ± 0.75	0.94 (.393)	2.75 ± 0.36	1.56 (.213)
	Bachelor’s degree	32.10 ± 4.57		57.86 ± 17.71		5.02 ± 0.79		2.88 ± 0.33	
	≥ Master’s degree	35.43 ± 4.79		65.36 ± 11.44		5.21 ± 0.75		2.91 ± 0.28	
Position	Staff nurse	31.86 ± 4.73	−2.25 (.026)	57.31 ± 18.42	−1.32 (.095)	4.98 ± 0.80	−1.05 (.297)	2.87 ± 0.33	0.01 (.999)
	Charge nurse	34.73 ± 4.93		63.73 ± 13.91		5.20 ± 0.49		2.87 ± 0.35	
Total clinical career (yr)^†^	< 5^a^	31.39 ± 4.67	3.46 (.033)	58.20 ± 18.06	0.20 (.820)	5.03 ± 0.76	0.49 (.613)	2.84 ± 0.34	1.45 (.236)
	5~9^b^	32.25 ± 5.12	a < c	58.53 ± 16.73		4.89 ± 0.91		2.95 ± 0.29	
	≥ 10^c^	33.67 ± 4.49		56.30 ± 19.94		5.03 ± 0.70		2.87 ± 0.37	
Current department career (yr)	< 2	32.54 ± 4.79	1.26 (.287)	57.23 ± 18.06	0.86 (.426)	4.91 ± 0.72	1.93 (.148)	2.85 ± 0.33	0.46 (.635)
	2~4	31.36 ± 5.22		56.65 ± 17.40		5.00 ± 0.86		2.87 ± 0.34	
	≥ 5	32.49 ± 3.87		61.29 ± 19.71		5.21 ± 0.74		2.91 ± 0.33	
Third-shift work	Yes	31.82 ± 4.89	−1.76 (.080)	57.71 ± 17.83	−0.20 (.840)	5.00 ± 0.79	−0.16 (.873)	2.88 ± 0.33	0.52 (.602)
	No	33.50 ± 4.06		58.45 ± 20.00		5.02 ± 0.76		2.84 ± 0.35	
Manual for delirium nursing care	Yes	32.56 ± 4.13	1.40 (.163)	56.31 ± 16.48	−1.22 (.226)	4.97 ± 0.75	−0.59 (.554)	2.90 ± 0.32	1.27 (.206)
	No	31.56 ± 5.44		59.61 ± 19.87		5.04 ± 0.82		2.84 ± 0.35	
Experience of education for delirium	Yes	33.01 ± 4.14	3.26 (.001)	56.56 ± 17.26	−1.17 (.242)	4.86 ± 0.74	−3.16 (.002)	2.91 ± 0.32	2.09 (.038)
	No	30.71 ± 5.39		59.77 ± 19.39		5.22 ± 0.79		2.81 ± 0.35	
Need for delirium education	Necessary	32.20 ± 4.76	1.95 (.052)	58.01 ± 18.12	0.87 (.384)	5.01 ± 0.78	0.89 (.372)	2.87 ± 0.33	−0.66 (.510)
	Not necessary	27.50 ± 4.93		50.00 ± 20.42		4.66 ± 0.80		2.98 ± 0.46	
Conflict with the caregiver of patient with delirium	Yes	32.20 ± 5.00	0.51 (.608)	58.20 ± 17.23	0.47 (.638)	4.95 ± 0.78	−1.51 (.132)	2.89 ± 0.33	1.54 (.126)
	No	31.79 ± 4.19		56.76 ± 20.76		5.15 ± 0.77		2.80 ± 0.33	

M = Mean; SD = Standard deviation.

^†^Post hoc = Duncan test.

**Table 4.** Correlations among Delirium Knowledge, Delirium Nursing-related Stress, Emotional Intelligence, and Delirium Nursing Competency

Variables	Delirium knowledge	Delirium nursing-related stress	Emotional intelligence	Delirium nursing competency
	r(*p*)			
Delirium nursing-related stress	.06 (.412)	1	-	-
Emotional intelligence	.02 (.769)	.01 (.913)	1	-
Delirium nursing competency	.24 (.001)	−.06 (.394)	.23 (.001)	1

**Table 5.** Factors influencing Delirium Nursing Competency

Variables	B	SE	β	t	*p*	VIF
(Constant)	1.85	0.23		8.19	< .001	
Experience of education about delirium (Ref. = No)	0.11	0.05	0.16	2.18	.031	1.14
Delirium knowledge	0.01	0.01	0.20	2.78	.006	1.07
Emotional intelligence	0.11	0.03	0.27	3.71	< .001	1.06
F = 7.18 (*p* < .001), R^2^ = 0.14, adj R^2^ = 0.12, Durbin-Watson = 1.94						

SE = Standard error; VIF = Variance inflation factor; Ref = Reference.

## Data Availability

The data that support the findings of this study are available from the corresponding author upon reasonable request.
